# Elevated Uptake of Plasma Macromolecules by Regions of Arterial Wall Predisposed to Plaque Instability in a Mouse Model

**DOI:** 10.1371/journal.pone.0115728

**Published:** 2014-12-22

**Authors:** Zahra Mohri, Ethan M. Rowland, Lindsey A. Clarke, Amalia De Luca, Véronique Peiffer, Rob Krams, Spencer J. Sherwin, Peter D. Weinberg

**Affiliations:** 1 Department of Bioengineering, Imperial College London, London, United Kingdom; 2 Department of Aeronautics, Imperial College London, London, United Kingdom; University of Amsterdam Academic Medical Center, Netherlands

## Abstract

Atherosclerosis may be triggered by an elevated net transport of lipid-carrying macromolecules from plasma into the arterial wall. We hypothesised that whether lesions are of the thin-cap fibroatheroma (TCFA) type or are less fatty and more fibrous depends on the degree of elevation of transport, with greater uptake leading to the former. We further hypothesised that the degree of elevation can depend on haemodynamic wall shear stress characteristics and nitric oxide synthesis. Placing a tapered cuff around the carotid artery of apolipoprotein E -/- mice modifies patterns of shear stress and eNOS expression, and triggers lesion development at the upstream and downstream cuff margins; upstream but not downstream lesions resemble the TCFA. We measured wall uptake of a macromolecular tracer in the carotid artery of C57bl/6 mice after cuff placement. Uptake was elevated in the regions that develop lesions in hyperlipidaemic mice and was significantly more elevated where plaques of the TCFA type develop. Computational simulations and effects of reversing the cuff orientation indicated a role for solid as well as fluid mechanical stresses. Inhibiting NO synthesis abolished the difference in uptake between the upstream and downstream sites. The data support the hypothesis that excessively elevated wall uptake of plasma macromolecules initiates the development of the TCFA, suggest that such uptake can result from solid and fluid mechanical stresses, and are consistent with a role for NO synthesis. Modification of wall transport properties might form the basis of novel methods for reducing plaque rupture.

## Introduction

The major cause of sudden death is coronary artery plaque rupture leading to thrombosis [1]. The lesion thought to undergo most ruptures has been termed the thin-cap fibroatheroma (TCFA) [2]. Biomechanical studies [3] and well-established effects of lipids on inflammatory and proteolytic processes [4], [5] are consistent with the large, lipid-rich core of this lesion playing a key role in its rupture. The association of the TCFA with elevated plasma cholesterol concentrations [2] suggests that the lipid in the core derives dominantly from circulating lipoproteins. Importantly, net uptake of such lipoproteins by the arterial wall depends not only on their plasma concentration but also on transport properties of the wall itself [6].

The aetiology of the TCFA is hard to study in people. Placing a tapered, flow-restricting cuff around the carotid artery of apolipoprotein E -/- (apoE -/-) mice induces the formation of plaques upstream and downstream of the cuff; those upstream of the cuff are rich in lipid and have a histological resemblance to the TCFA, whereas those downstream of the cuff contain less lipid and resemble stable lesions [7]. Theoretical predictions suggest that both regions are characterised by relatively low shear stress, but that the shear is more oscillatory in the downstream area [8]. The cuff also leads to a non-uniform expression of endothelial nitric oxide synthase (eNOS) [8]. These findings provide a model for investigating the TCFA, imply that haemodynamic stresses are important in its pathogenesis, and are consistent with a key role for the NO signalling pathway.

Uptake of circulating macromolecules by the aortic wall of the mature rabbit is highly non-uniform [9], [10]; its pattern depends on blood flow [11] and NO synthesis [12], [13], and correlates spatially with the subsequent occurrence of spontaneous [14] and diet-induced [13], [15] lipid deposition. Here we test the hypothesis that, prior to the development of lesions in the cuffed mouse carotid, macromolecule uptake is elevated in both regions prone to lesion development, is sensitive to flow and NO synthesis, and is higher upstream than downstream of the cuff, thereby providing a plausible mechanism for why the upstream rather than the downstream lesions most closely resemble the TCFA.

## Methods

### Tracer preparation

Bovine serum albumin (fatty acid free, Fisher Scientific, UK) was labelled with sulphorhodamine acid chloride (Lissamine rhodamine, SigmaAldrich, UK) and purified of low molecular weight fluorescent material as previously described [16]. The tracer has physical and chemical properties closely resembling those of the unlabelled protein and is stable *in vitro* and *in vivo* [17].

### Flow-modifying cuff

Rigid, polyether ketone perivascular cuffs (Promolding, the Netherlands) were made in two halves so that they could be placed around the vessel [8]. When assembled, their lumen narrowed linearly from 500 to 250 µm in diameter over their length of 1.5 mm.

### Cuff placement

All animal experiments complied with the Animals (Scientific Procedures) Act 1986 and were approved by the Local Ethical Review Process Committee of Imperial College London (Home Office Licence PPL 70/7333). Cuffs were assembled around the left common carotid artery of male C57bl/6 mice aged 8–10 weeks (Charles River, UK) under anaesthesia with 1.5–2.5% isofluorane and 2% O_2_, as previously described [7], [8]. Anaesthetic depth was assessed from respiratory rate, hind limb muscle tone and pedal withdrawal reflex. The cuff was placed in the orientation used in previous studies (wide end upstream) or, to further elucidate the roles of mechanical stresses, in the reverse orientation (narrow end upstream). The wound was sutured and of buprenorphine (Centaur, UK; 0.05 mg/kg *sc*) was injected for pain relief.

### Tracer uptake studies

Tracer (150 mg/kg) was introduced via a tail vein into the circulation of conscious mice one week after cuff placement, by which time they would have reached>90% of their maximum size [18]. Non-cuffed mice were used as a control. The tracer was allowed to circulate for 10 minutes. It was omitted in some mice to allow assessment of tissue autofluorescence in the tracer channel. To inhibit NO production, L-*N* ^G^-nitroarginine methyl ester (L-NAME, SigmaAldrich, UK; 50 mg/kg *ip*) was administered 30 minutes before tracer in some animals.

Mice were killed by an overdose of pentobarbitone (Euthatal, Centaur, UK; 160 mg/kg *ip*) and, following thoracotomy, the left ventricle was cannulated so that the cardiovascular system could be flushed with approximately 5 mL of physiological saline and fixed *in situ* for 30 minutes with 15% formaldehyde (SigmaAldrich, UK) from a reservoir 100 cm above the animal. The interval between death and flushing was approximately 3 minutes. In some animals, tracer was injected just before death in order to determine whether uptake in this 3-minute interval had an influence on the results. For all animals, both common carotid arteries were excised and stored in fixative at 4°C.

### Imaging tracer uptake by confocal microscopy

Each carotid artery was divided lengthwise into two pieces which were mounted luminal side downwards in Vectashield (Vector Labs, UK) on coverslips and imaged *en face* with an inverted Leica SP5 laser scanning confocal microscope as previously described [16], with minor modifications as detailed below. The use of a ×20 0.7NA immersion objective in conjunction with tile scanning gave high spatial resolution (limited by the pixel size of 3×3 µm) over a wide field of view (∼0.5×5 mm). Tracer fluorescence (excitation 561 nm, emission 585–620 nm) and tissue autofluorescence (excitation 458 nm, emission 456–515 nm) were imaged, keeping all instrument settings identical between experiments.

### Image processing

Confocal image stacks were analysed by an in-house MATLAB (MathWorks, v7.7.0) programme. The luminal surface was detected by thresholding the autofluorescence channel stacks. Then the maximum intensity between 0–25 µm into the wall (z-direction) from the luminal surface was obtained at each x-y location. This method was chosen in preference to our earlier method, based on summation of intensities over a fixed distance into the wall, because foci of very high autofluorescence intensities, and consequent glare, occurring at cuff margins gave rise to errors in locating the luminal surface; use of the maximum projection consistently found the highest tracer concentration within the wall. Spatial biases in sensitivity in the x-y plane were corrected using images of a uniformly fluorescing slide; the falloff in intensity in the z-direction was corrected using calibration data obtained previously [16]. Temporal fluctuations were assumed to average out over the large number and long duration of imaging sessions that were used. Intensities were averaged circumferentially for each lengthwise location along the carotid. Data for the two segments from each carotid were averaged prior to the calculation of a mean for each group of mice.

### Vascular corrosion casting

Corrosion casts were used to assess the change in geometry produced by the cuff. A cannula, made from a 26G butterfly and filled with saline, was inserted into the left ventricle of euthanized mice and secured with cyanoacrylate glue. The cannula was then attached to a syringe containing methyl methacrylate casting material (Batson's No. 17, Polysciences, Inc., Germany, prepared according to the manufacturer's instructions) and to a manometer, which allowed the infusion pressure to be monitored. Casting material (2–3 mL) was injected at a pressure of 95–100 mmHg into the vascular system until hard and the preparation was left to cure completely overnight. The carcase was then placed in a 30% w/v potassium hydroxide solution for 5 days to corrode the tissue. The cast was cleaned with detergent (DECON) for 24 h before use.

### MicroCT scanning and geometric reconstruction

Casts were scanned by microCT (Metris X-Tek HMX-ST). The resulting DICOM images had an isotropic voxel size of 5.5 µm. They were segmented (Amira 5.2.2, Visage Imaging, Inc.) and a surface definition was extracted and smoothed (Amira; VMTK, www.vmtk.org; Gambit 2.4.6, ANSYS, Inc.). Following computation of the vessel centreline with VMTK, cross sectional area and Shape Index were computed at 5 µm intervals along the vessel length with a purpose-written VTK-Python script (www.vtk.org). Shape index [18] is defined as 4πA/(P^2^), where A = cross-sectional area and P = perimeter; it has a value of 1 for a circle and 0 for a line.

### Doppler ultrasound measurements of blood flow

Flow was assessed one week after cuff placement, and in control animals that had not undergone surgery. Animals, anaesthetised as described above, were placed supine on an imaging platform. Heart rate, temperature, and electrocardiogram were recorded. Neck hair was removed and a pre-warmed gel was liberally applied to the skin to acoustically couple the scan head. A 30 MHz mechanical transducer (RMV-707B) in a mechanical holder, coupled to a Vevo770 ultrasound system (VisualSonics, Canada), was placed in contact with the gel. B-mode was used to align the transducer with the long axis of the vessel and to locate regions of interest, before switching to Power Doppler mode for measurements of blood flow velocity. The insonation angle averaged 58°±1.7°. Results were analysed using VisualSonics software. The programme identified the maximum frequency (and hence maximum velocity) of the spectrum at each timepoint in the trace. With user input to define the relevant parts of the cardiac cycle, it then calculated peak, end-diastolic and cycle-averaged maximum velocities. Each of these results was averaged over 10 contiguous heart beats (approx. 1 breathing cycle) in each trace.

### Computational Fluid Dynamics

Steady flow was simulated for one forward and one reverse cuff geometry with an in-house spectral/hp element solver [20]. Using VMTK, cylindrical flow extensions were added to the bifurcation outflows, to facilitate the application of boundary conditions, and the surface was remeshed according to a size function derived from the distance of the surface to the vessel centreline, so that the size of elements decreased as the vessel narrowed. A volume mesh of tetrahedral and prismatic boundary layer elements was generated using Gambit and TGrid (ANSYS, Inc); external faces of the volume elements were curved as previously described [19]. The volume meshes for the forward and reverse cuff geometries contained, respectively, 25,215 and 21,912 tetrahedral elements and 7,992 and 6,834 prismatic elements.

To estimate inlet conditions for a conscious resting mouse, ultrasound-derived velocities were multiplied by factors of 1.05 and 1.72, corresponding to the minimum and maximum effects of anaesthesia [22]. To estimate inlet conditions in active rather than resting mice, velocities were scaled by a further factor of 1.88 [22]. Inlet flow was assumed to be fully developed and flow splits at the carotid bifurcation were computed using Murray's law. A fully developed outflow was applied to one branch and a zero velocity gradient boundary was imposed at the outflow of the other. Blood was assumed to be Newtonian and incompressible with a viscosity of 0.004 kg/(m.s) and density of 1015 kg/m^3^; arterial walls were assumed to be rigid, and all velocity components were set to zero along them. A 7^th^ order polynomial expansion was used to represent the solution in each element. (The total wall force changed by <2% when switching from 7^th^ to 9^th^ order solutions).

Wall shear stress and wall pressure were averaged circumferentially along the length of the vessel by mapping the mesh surface onto a rectangular parametric space in VMTK and averaging nodal quantities across bands 40 µm long. Minimum wall shear was computed by taking the mean of the lower 5^th^ percentile of shear values within each band. A reference pressure (the value obtained 2 and 3 mm downstream of the throat in the forward and reverse geometries, respectively) was subtracted from each set of mean pressures to better compare results obtained under different inlet conditions.

### Statistics

Student's paired t-test was used to assess differences in uptake between left and right carotids or between upstream and downstream cuff margins (mean values being taken over a width of 0.7 mm centred on peak uptake). Student's unpaired t-test was used for comparisons where different conditions were examined in different mice; in these cases, which concerned uncuffed arteries, data were averaged along each vessel to obtain a mean value. P<0.05 was taken as the criterion of significance.

## Results

### The cuff induced a severe stenosis

**Figure 1:**
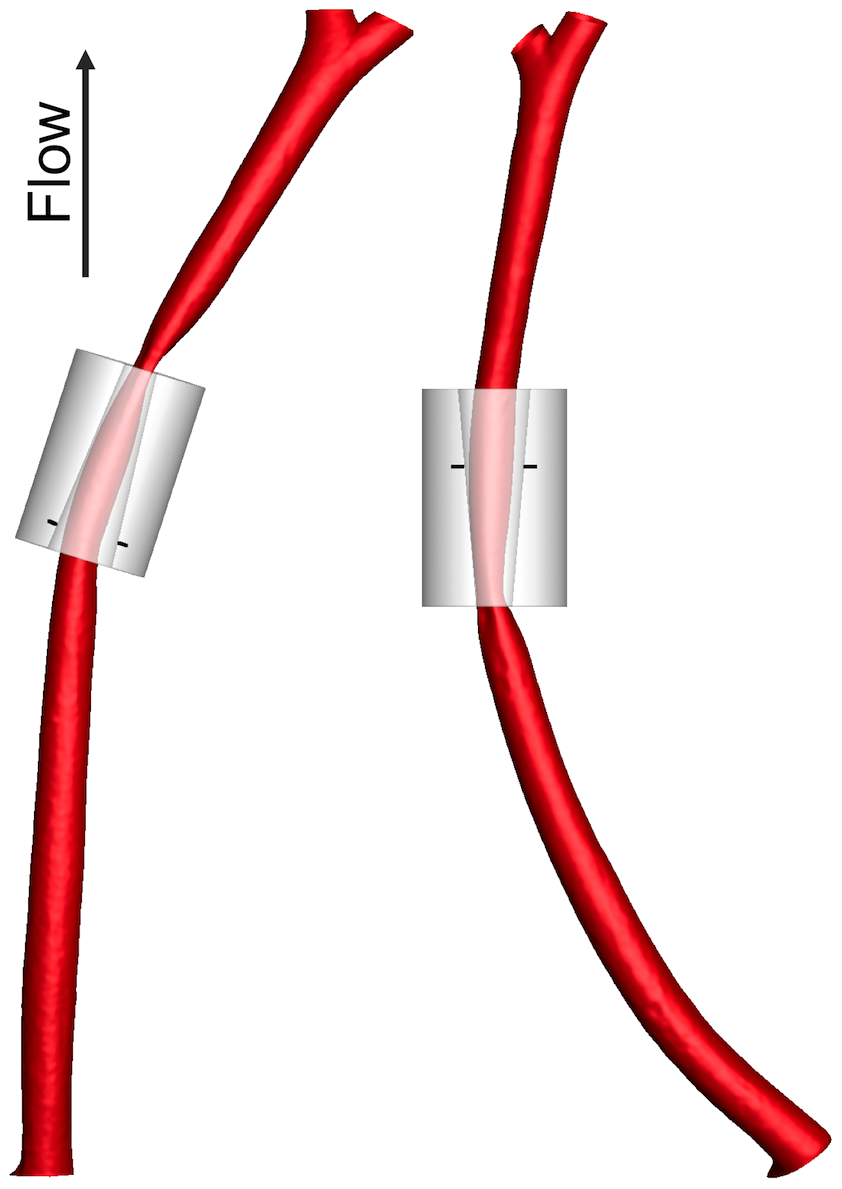
Reconstruction of cuffed vessels. Reconstruction of the luminal geometry of the left carotid artery from two mice, one with the cuff in the conventional direction (left) and one with it reversed (right). The digital model of the cuff was fitted onto the reconstruction of the corrosion cast by aligning its narrow end with the most restricted point on the cast, and its long axis with the line connecting this point and a point on the cast's centreline 1.5 mm upstream or downstream for the conventional or reversed cuff orientations, respectively. Marks indicate where the wall would first come into contact with the forward cuff or would last be in contact with the reversed cuff, assuming wall thickness is 10% of luminal diameter.

Fig. 1 shows reconstructions of the carotid lumen with the cuff superimposed. The wide end of the cuff was too wide to be in contact with the outer surface of the vessel (allowing for a wall thickness of 10% of the luminal diameter and 10% resin shrinkage during setting), meaning that there was no abrupt narrowing at the upstream end with the cuff in the conventional orientation, and no sharply diverging region at the downstream end with the cuff in the reversed direction.

**Figure 2:**
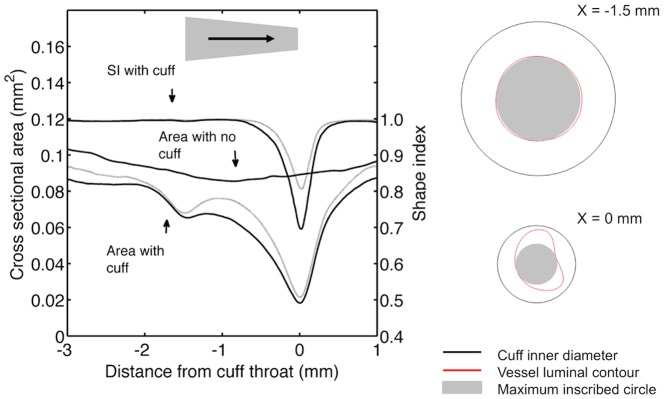
Geometry of cuffed vessels. Left. Cross sectional area and shape index of the left carotid lumen in mice with the cuff in the conventional direction. The location and orientation of the cuff with respect to the x-axis are shown by the shaded area; the arrow indicates the mean flow direction. Shape index = 1 for a circle and 0 for a line. Mean (dark line) +1 SEM (light line), n = 3. For comparison, the cross sectional area of one uncuffed left carotid is also shown. Right. Cross sections at the upstream (x = −1.5 mm) and downstream (x = 0 mm) ends of the cuff, showing the cuff inner diameter, the luminal contour of the vessel, and the maximum inscribed circle that can be fitted within the lumen.

**Figure 3:**
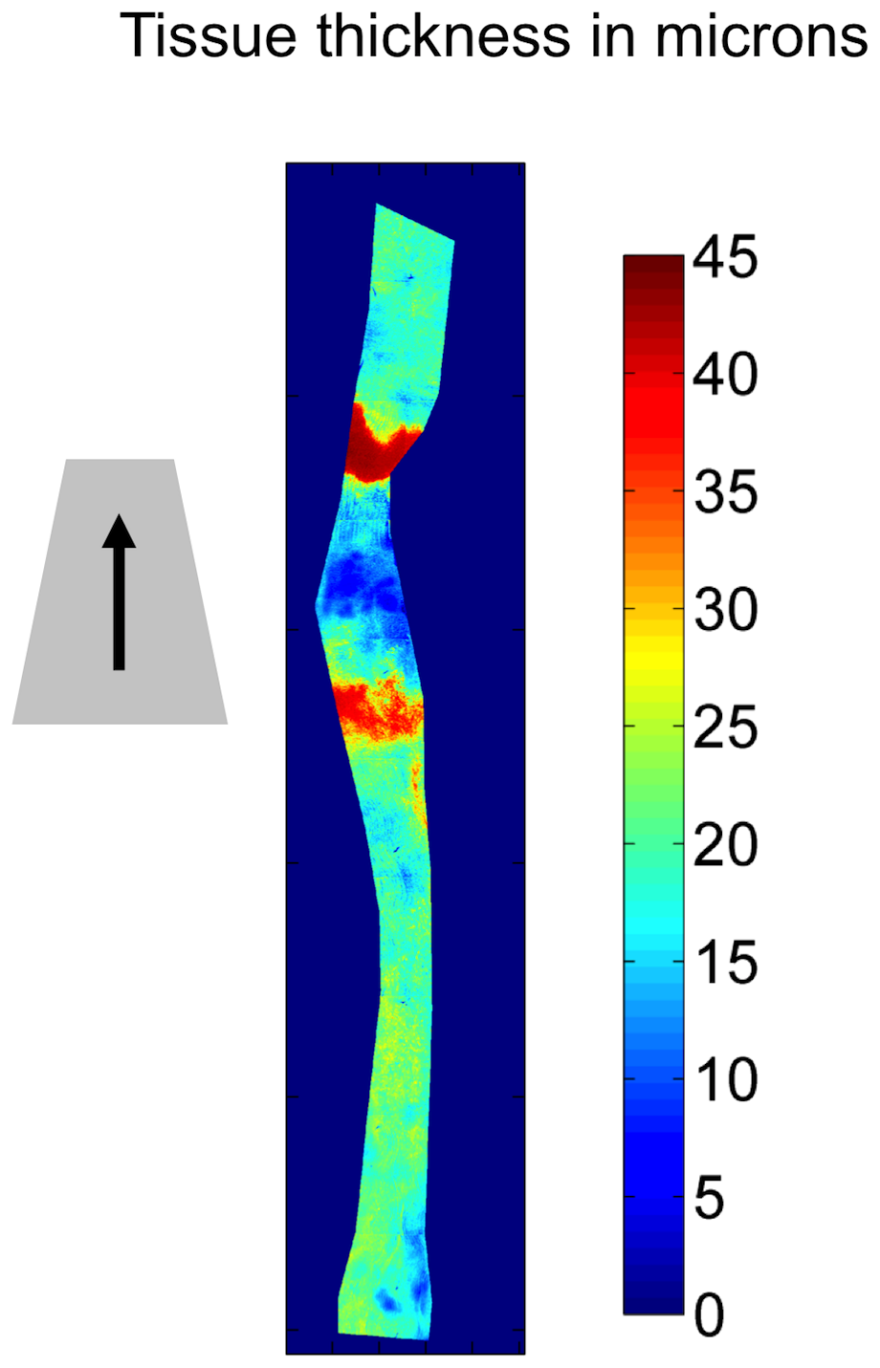
Effect of the cuff on wall thickness. Thickness of the wall of a mouse carotid artery, obtained from *en face* stacks of confocal images, 1 week after a cuff was placed around the vessel in the conventional direction. The vessel was divided longitudinally; the image shows one half of it. Flow direction, cuff location and cuff orientation are indicated as described for Fig. 2. Compared to uncuffed regions, the wall was thickened near the throat of the cuff and where the wide end of the cuff lost contact with the vessel, and it was thinned within the cuffed region.

Fig. 2 shows morphological data derived from the reconstructions of the conventionally-oriented cuff. The stenosis is readily apparent. It is also clear that the luminal cross section of the vessel was highly non-circular at the stenosis. The reduction in cross-sectional area at the wider, upstream end of the cuff is consistent with remodelling of the wall in response to the change in mechanical properties. This view is also supported by measurements showing an increase in wall thickness (Fig. 3) and tissue autofluorescence (see below) at this location.

Thinning of the vessel wall within the cuff is also apparent in Fig. 3. Similar thinning is evident at points of high wall curvature in previously-published sections of cuffed mouse carotids [23]; it has also been observed in cuffed rabbit aortas [24].

### The stenosis restricted flow and caused a jet

**Figure 4:**
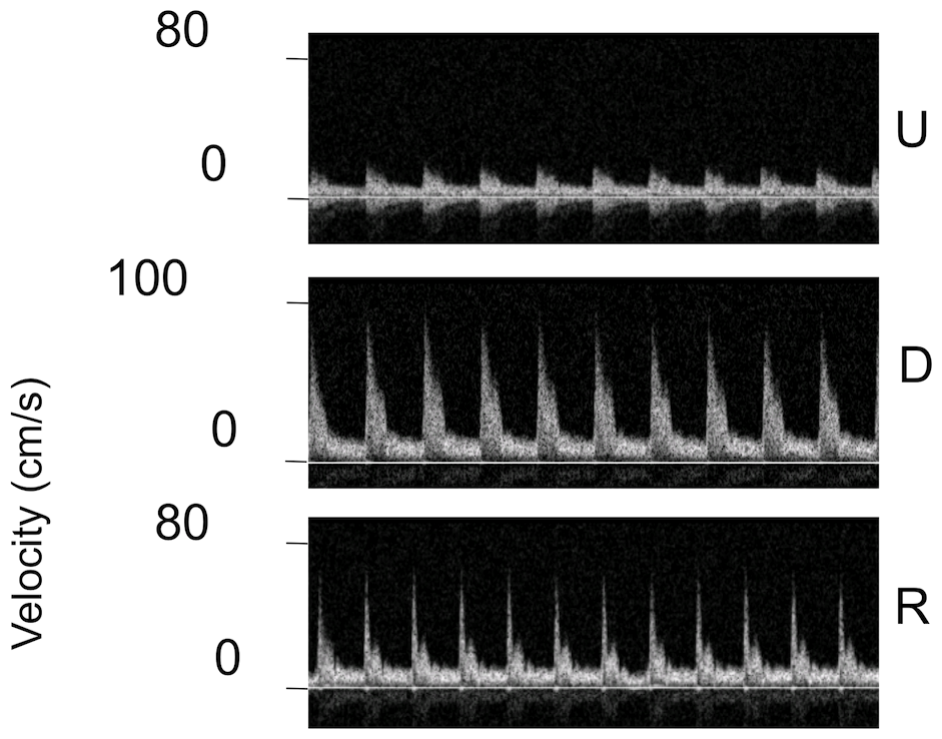
Measured blood flow velocities. Doppler ultrasound traces showing flow velocity versus time at the upstream (U) and downstream (D) ends of the cuffed left carotid and in the uncuffed right carotid (R) of a mouse with the cuff in the conventional orientation. Note the altered y-axis scale for D.

Doppler ultrasound traces, indicating blood flow velocities as a function of time, are shown for a carotid artery with the cuff in the conventional direction and for the contralateral control in Fig. 4. Numerical values for the data, and also for a cuff in the reversed direction and for an uncuffed mouse, are given in Table 1. As expected, velocity was reduced upstream of the cuff compared to the control vessel (by about 50%, for the cardiac cycle-average value), and was elevated downstream of the cuff (the downstream average being triple the upstream one), presumably due to the formation of a jet. Further downstream, the velocity was again reduced (data not shown). Flow could not be measured within the cuff, which is opaque to ultrasound. With the cuff in the reverse orientation, cycle-average upstream flow was again halved compared to the contralateral carotid, but there was no evidence for a jet downstream of the cuff (average velocities being increased by <50%). Without a cuff, velocities were approximately equal in right and left carotids (mean velocity 10.9 and 12.9 cm/s respectively).

**Table 1:** Blood flow velocities measured conventional and reversed cuff.

	Forward Cuff			Reverse Cuff		
	LC-U	LC-D	RC	LC-U	LC-D	RC
Mean	7.4	20.2	14.0	5.5	7.9	11.9
Peak	13.6	53.1	47.0	17.8	24.6	29.0
Diastole	4.5	9.4	8.4	2.1	5.5	5.8

Mean, peak and diastolic blood flow velocities in cm/s, measured by Doppler ultrasound, with the cuff in the conventional and reversed orientation. Velocity was measured at the upstream (“LC-U”) and downstream (“LC-D”) ends of the cuff in the left carotid, and in the uncuffed right carotid (“RC”). Values are averages for ten cardiac cycles.

### The cuff modified patterns of wall shear stress and pressure

**Figure 5:**
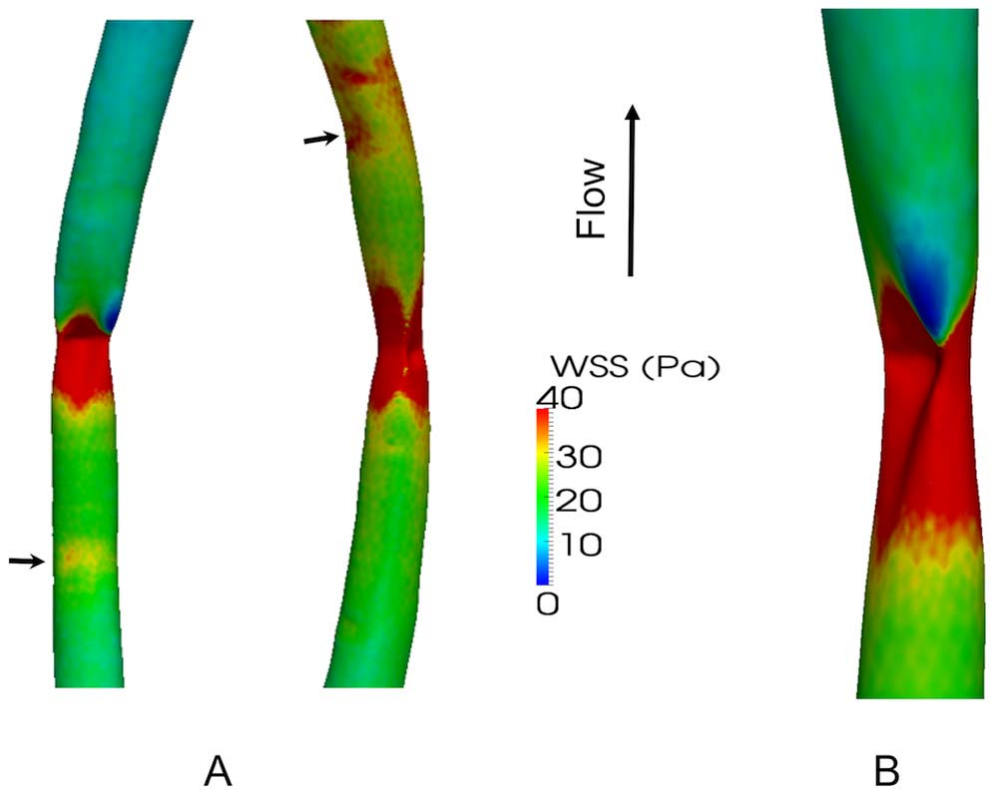
Maps of hemodynamic wall shear stress (WSS) in cuffed vessels. A. Colour maps of WSS, obtained by CFD, in carotid arteries of two mice, one with the cuff in the conventional direction (left) and one with it reversed. The throat of the cuff occurs at the narrowest part of the reconstruction; the position of the wide end of the cuff is indicated by an arrow. B. Detail of the throat region with the cuff in the conventional direction.

**Figure 6:**
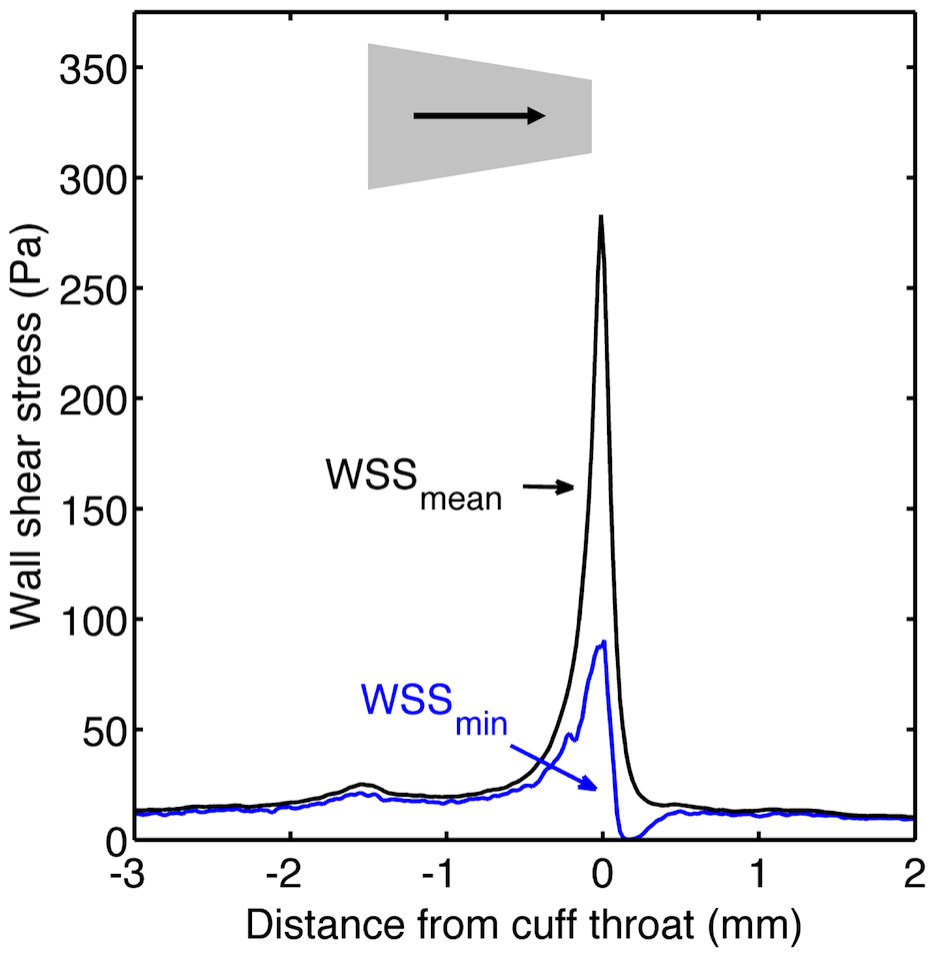
Mean and minimum WSS along vessels with the cuff in the conventional orientation. Plot of circumferential minimum and circumferential mean WSS versus distance along the vessel for the mouse with the cuff in the forward direction. Flow direction, cuff location and cuff orientation are indicated as described for Fig 2.

**Figure 7:**
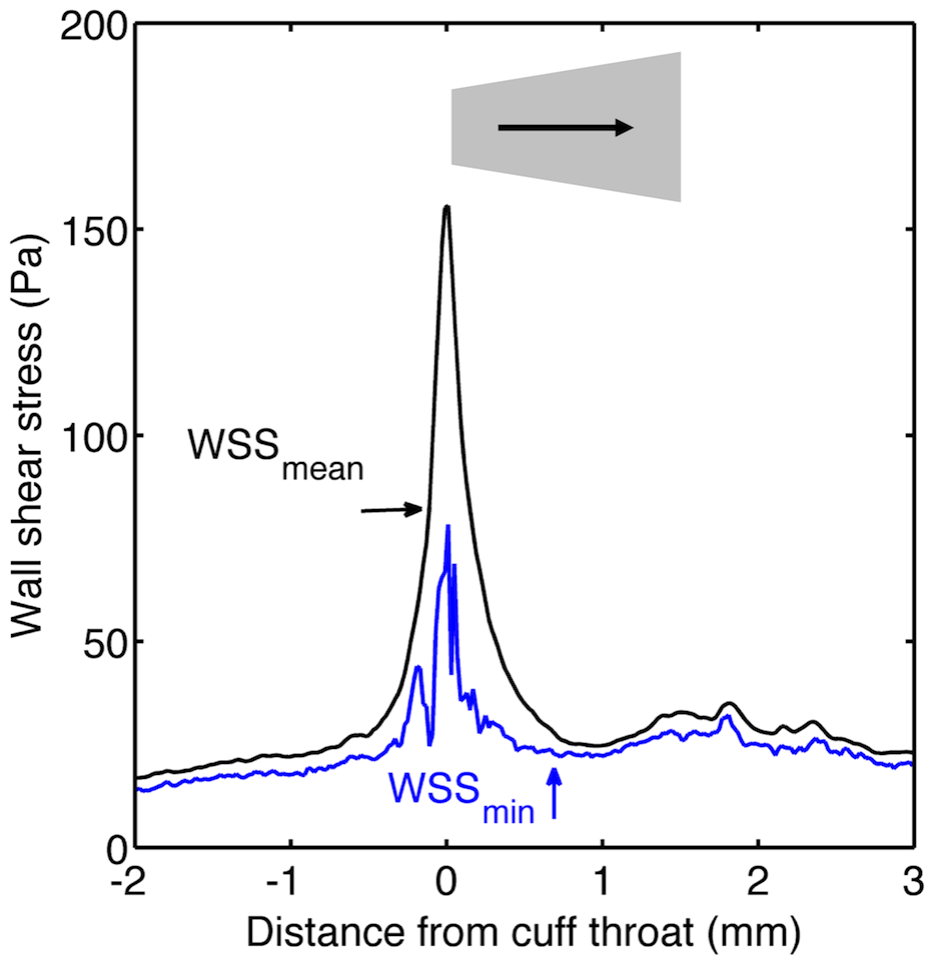
Mean and minimum WSS along vessels with the cuff in the reverse orientation. Plot equivalent to Fig. 6 but for the mouse with the cuff in the reverse direction.

Fig. 5A maps the pattern of wall shear stress computed for cuffs in the conventional (forward) and reverse directions, respectively. Fig. 5B shows shear stress in the vicinity of the throat in more detail. Figs. 6 and 7 plot the corresponding circumferential minimum and mean shear, respectively, at each point along the carotid. Wall shear stresses upstream of the cuff were approximately 10 Pa. That is an order of magnitude higher than in the human carotid [25], despite the presence of the flow-limiting stenosis, reflecting the established inverse relation between shear and body weight [26], [27]. The maximum value induced artificially by the cuff exceeded 300 Pa, which is approximately five-fold higher than naturally-occurring peak values previously computed for the mouse aorta [28].

The data for these figures were obtained assuming a cross-sectionally averaged systolic peak inflow velocity of 12 cm/s, equivalent to a Reynolds number (Re) of 11. Table S1 in S1 File gives other values of flow velocity and Re for which simulations were performed, and Figures S1A-D in S1 File give the corresponding maps of wall shear stress. Flow patterns computed for the cuff in the forward orientation were highly dependent on inflow velocity. At the lower velocities, flow accelerated with increasing distance down the cuff, as the walls of the cuff converged, and then decelerated smoothly once past the throat, as the vessel increased in cross-sectional area. In contrast, at inflow velocities ≥12 cm/s (Re≥11), the flow separated in a bubble downstream of the throat over part of the vessel circumference. The recirculation region, characterised by reverse flow and low wall shear stress, was surrounded by a stagnation region. The flow velocity at which recirculation was first observed is above that recorded in anaesthetised mice (and also above that expected in resting mice using conservative estimates of the effect of anaesthesia on flow), but within the range of velocities expected for conscious, active mice.

The size of the recirculation region increased with increasing inflow velocity. Since velocities vary during the cardiac cycle, this result implies that there will be areas of the wall which experience forward and reverse flow (i.e. oscillatory shear stress) during each heartbeat. The oscillatory shear region is likely to be small because even at the highest plausible flows the recirculation zone was only around 250 µm long and occupied only about 1/4 of the circumference of the vessel.

Flow patterns simulated for the reversed cuff were less dependent on velocity. Flow accelerated sharply at the throat and then decelerated gradually with distance down the cuff for all values of Re. The divergence of the cuff walls was sufficiently gradual that there was no separation at even the highest inflow velocities.

For both orientations, there was a small, transient increase in shear at the wide end of the cuff that may be explained by the narrowing of the vessel lumen seen at this location (see above).

**Figure 8:**
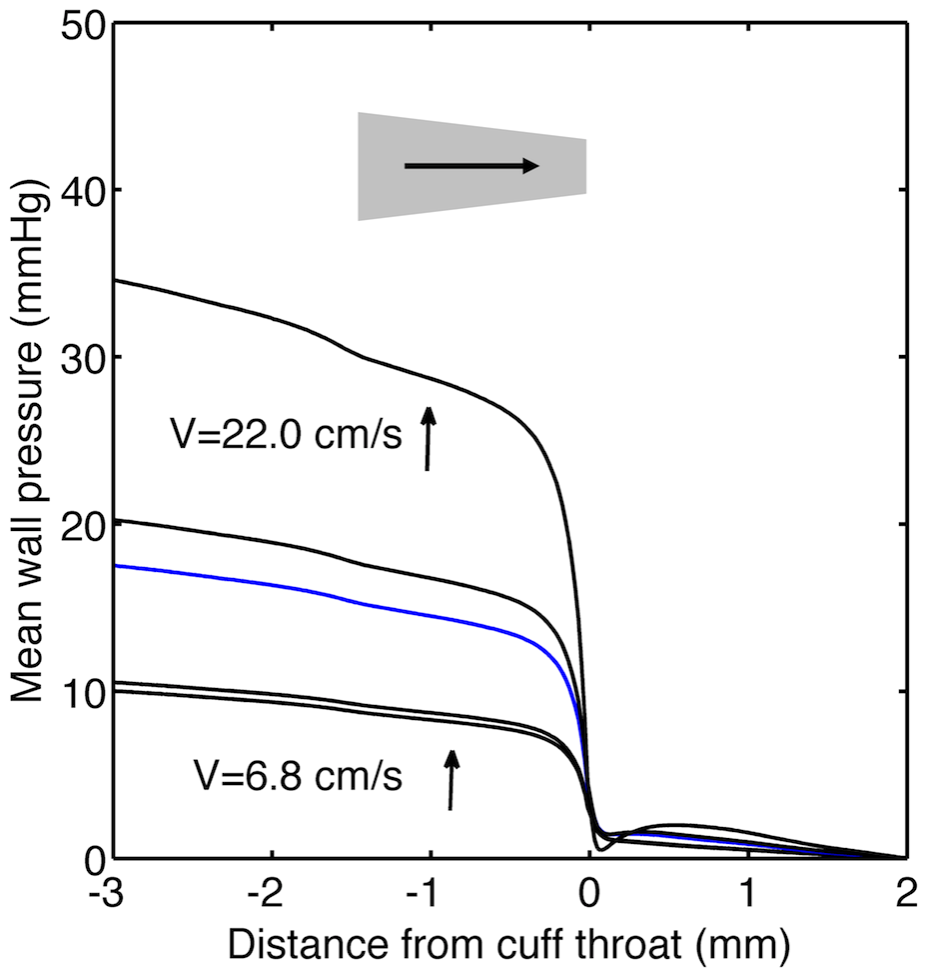
Pressure drop in cuffed vessels. Plot of pressure at the arterial wall versus distance down the carotid of a mouse with the cuff in the conventional direction. Flow direction, cuff location and cuff orientation are indicated as described for Fig. 2. Pressure is expressed relative to the pressure 2 mm downstream of the end of the cuff. Values were derived from CFD simulations that used the different inflow velocities shown in the first row of Table S1 in S1 File. The blue line is the case for v = 12 cm/s.

Pressure fell throughout the conventionally-oriented cuff but the rate of decrease increased substantially in the throat (Fig. 8). The pressure drop in the throat was greater at higher inflow velocities. Although such pressure drops might be expected from the acceleration of the flow, there was almost no pressure recovery after the throat, reflecting the low Reynolds numbers and hence high viscous losses, which are neglected by Bernoulli's principle. (Appendix S1 in S2 File gives a more comprehensive discussion of the pressure losses and their concordance with previous investigations at stenoses). Note that transmural pressure may vary not only because of these changes in luminal pressure but also as a result of patchy contact between the wall and the cuff.

### Tracer fluorescence was independent of position and side in uncuffed vessels

**Figure 9:**
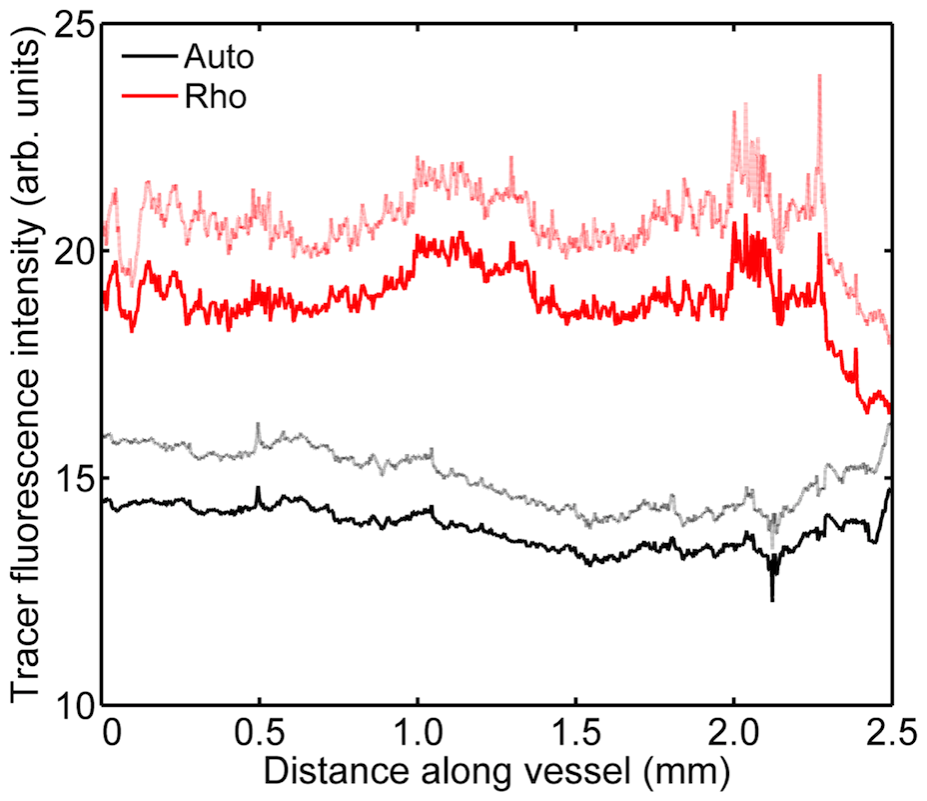
Tracer uptake along vessels in uncuffed mice. Fluorescence intensity along the length of carotid arteries of mice without cuffs; data from left and right carotids have been combined. Rhodamine-labelled tracer was administered to the mice (“Rho”) or was omitted to assess autofluorescence (“Auto”). Mean (dark line) +1 SEM (light line), n = 4.

In uncuffed animals receiving tracer, there were no substantial variations in fluorescence along either the left or the right carotid; the largest coefficient of variation for a single vessel was 0.19. Mean intensities were not significantly different between the two vessels (P = 0.24), and substantially greater than the autofluorescence seen in uncuffed animals that did not receive tracer (Fig. 9).

### Tracer uptake was modified by cuff placement

**Figure 10:**
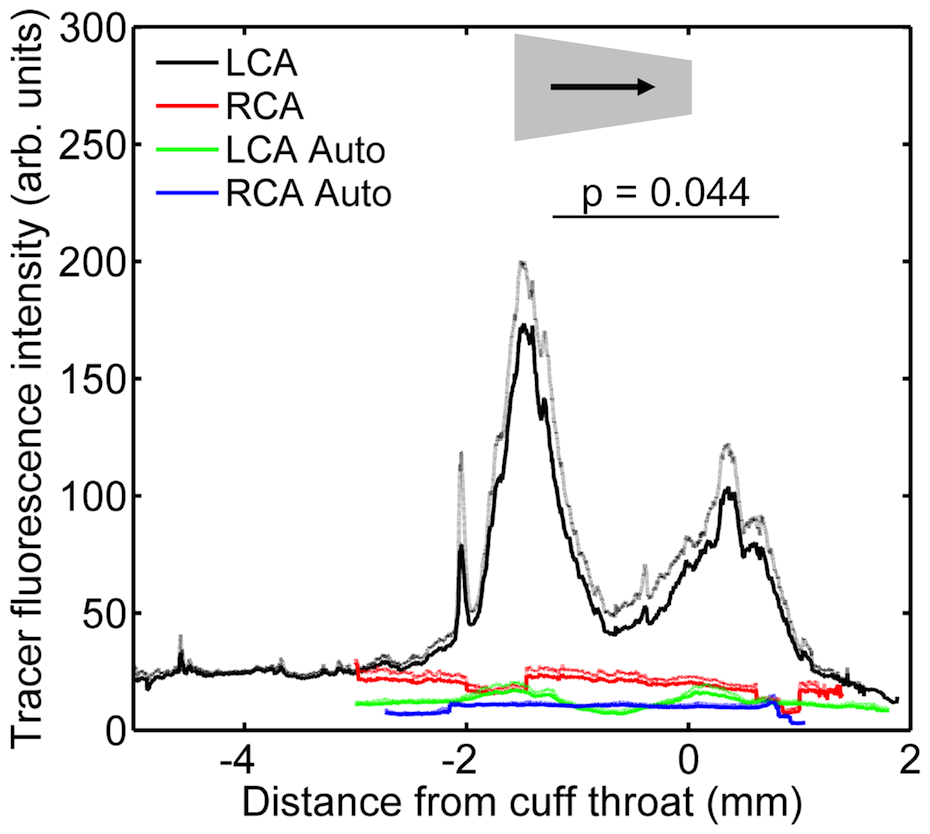
Tracer uptake along vessels with the cuff in the conventional orientation. Fluorescence intensity along the length of carotid arteries of mice having the cuff placed around their left carotid in the conventional direction. Intensity is shown separately for the cuffed left carotid (“LCA”) and uncuffed right carotid (“RCA”) of mice administered tracer or not administered tracer (indicating autofluorescence; “Auto”). Flow direction, cuff location and cuff orientation are indicated as described for Fig. 2. Mean (dark lines) + 1 SEM (light lines), n = 10 for mice given tracer and n = 3 for autofluorescence.

In animals where a cuff was placed in the conventional direction (narrow end downstream), fluorescence was again uniform along the length of the uncuffed contralateral carotid and higher than the autofluorescence seen in equivalent vessels from animals not administered tracer (Fig. 10).

In the cuffed carotids, fluorescence (Fig. 10) was unchanged far upstream of the cuff, and only slightly elevated within the central part of the cuff itself, compared to the contralateral control vessel. However, there were two peaks of fluorescence, each having a length approximately equal to the length of the cuff, where intensities were elevated many-fold. The upstream peak was centred on the upstream margin of the cuff and the downstream peak was centred approximately 0.5 mm downstream of the cuff. The upstream peak was greater than the downstream peak. These raised intensities are interpreted as increases in uptake rather than increases in autofluorescence because data from animals not administered tracer showed that autofluorescence was much less elevated in the vicinity of the cuff margins. Analysis of experimental values after subtraction of autofluorescence values confirmed that uptake was significantly higher in the upstream than the downstream peak (P = 0.044).

### Cuff reversal did not reverse the pattern of uptake

**Figure 11:**
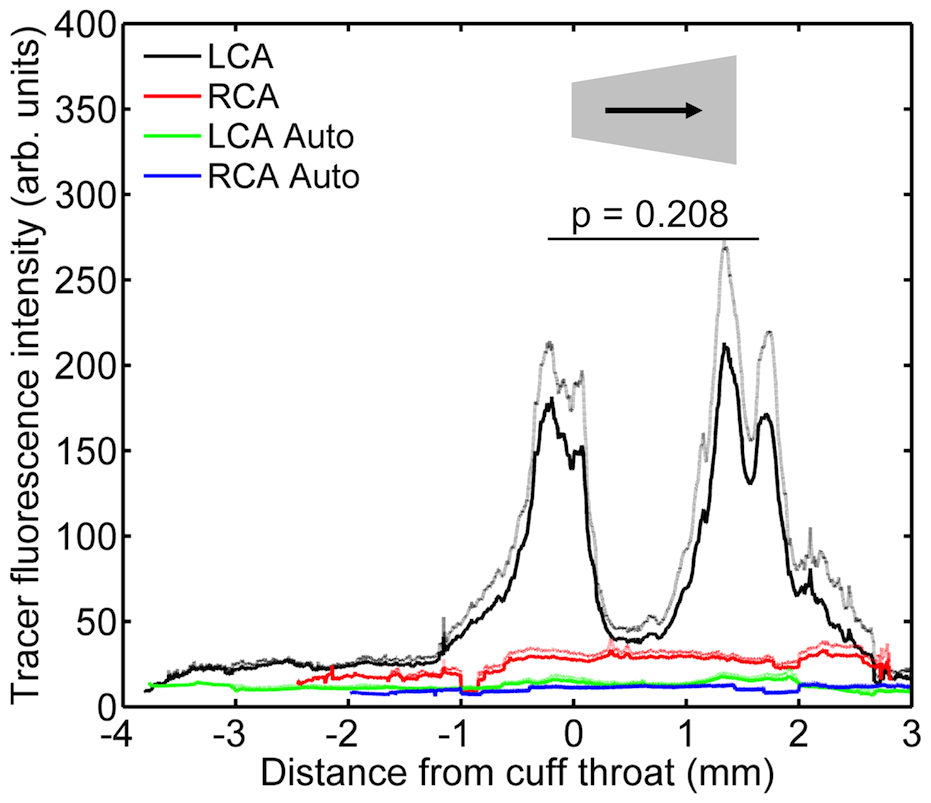
Tracer uptake along vessels along vessels with the cuff in the reverse orientation. A plot equivalent to Fig. 10 but for mice with the cuff in the reverse direction; n = 8 for tracer and n = 7 for autofluorescence.

Reversal of the cuff direction had no substantial effect on tracer fluorescence in the contralateral carotid, or on autofluorescence in either the cuffed or the contralateral vessel (Fig. 11), compared to the previous experiment. In the cuffed carotid exposed to tracer (Fig. 11), fluorescence was again not elevated in regions upstream of the cuff or within the central part of the cuff itself, and there were two peaks broadly coinciding with the ends of the cuff. However, there was no significant difference between the two peaks (P = 0.208 after subtraction of autofluorescence). Thus the ratio of the two peaks was neither the same nor the opposite of that seen with the cuff in the conventional orientation.

### Inhibition of eNOS modified tracer uptake

**Figure 12:**
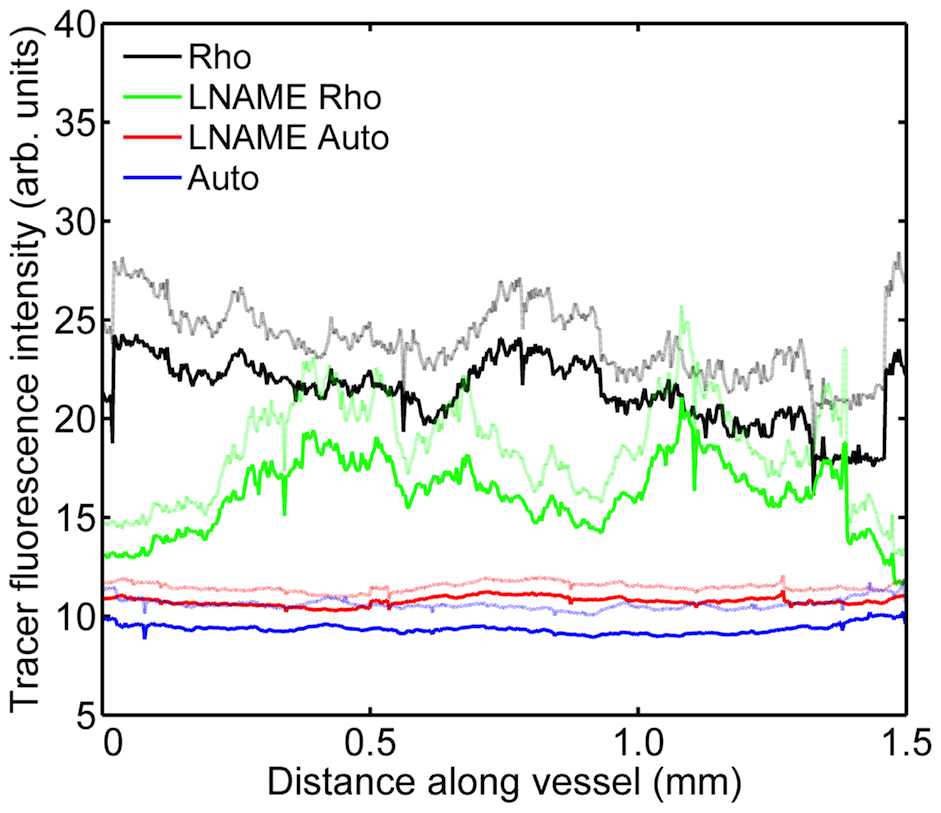
Effect of L-NAME on tracer uptake along vessels in uncuffed mice. Fluorescence intensity along the length of carotid arteries of mice without cuffs; data from left and right carotids have been combined. Mice were administered the rhodamine-labelled tracer with (“LNAME Rho”) or without (“Rho”) the NO synthase inhibitor L-NAME. Autofluorescence was assessed in mice not administered the tracer, again with (“LNAME Auto”) or without (“Auto”) the inhibitor. Mean (dark lines) + 1 SEM (light lines), n = 3-4 per group.

L-NAME visibly constricted superficial veins. In animals not undergoing surgery, for which left and right carotid values were averaged, the mean level of fluorescence was substantially reduced by L-NAME (Fig. 12); after subtraction of appropriate autofluorescence values, treated and untreated values differed by a factor of 2 (p = 0.0496). Indeed, fluorescence intensities in some animals receiving tracer and L-NAME were not higher than autofluorescence (which remained broadly unchanged), implying negligible uptake.

**Figure 13:**
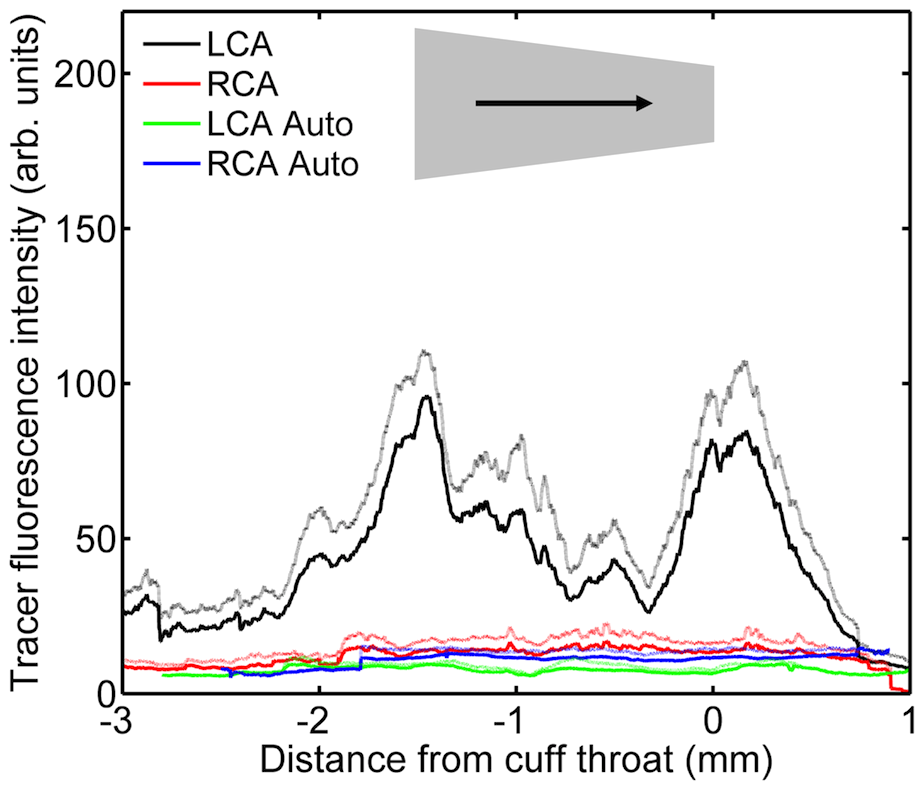
Effect of L-NAME on tracer uptake along vessels with the cuff in the conventional orientation. Fluorescence intensity along the length of carotid arteries of L-NAME-treated mice having the cuff placed around their left carotid in the conventional direction. Intensity is shown separately for the cuffed left carotid (“LCA”) and uncuffed right carotid (“RCA”) of mice administered tracer or not administered tracer (indicating autofluorescence; “Auto”). Flow direction, cuff location and cuff orientation are indicated as described for Fig. 2. Mean (dark lines) +1 SEM (light lines), n = 6 for mice given tracer and n = 2 for autofluorescence.

The visible difference between upstream and downstream peaks of fluorescence intensity in animals that had the cuff in the conventional direction and received tracer but no L-NAME (Fig. 10) was completely absent in the plot for animals additionally administered L-NAME (Fig. 13). After subtraction of appropriate autofluorescence values, there was no significant difference in peak height for the latter group (p = 0.599), unlike the former.

### The effect of post mortem uptake was negligible

**Figure 14:**
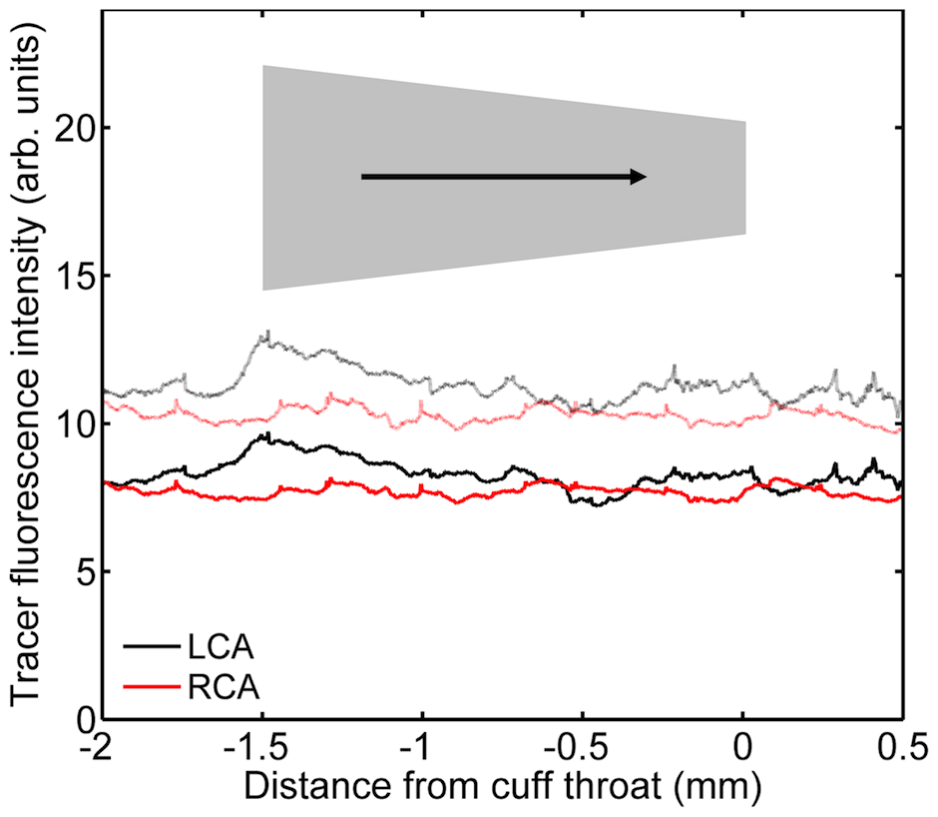
Tracer uptake along vessels in mice administered tracer just before death. Fluorescence intensity along the length of carotid arteries of mice having the cuff placed in the conventional direction and administered tracer 1 minute rather than 10 minutes before sacrifice. Intensity is shown separately for the cuffed left carotid (“LCA”) and uncuffed right carotid (“RCA”). Flow direction, cuff location and cuff orientation are indicated as described for Fig. 2. Mean (dark lines) +1 SEM (light lines), n = 3.

The patterns shown in Figs. 9–13 did not arise from uptake occurring in the interval between death and flushing tracer from the arterial lumen because fluorescence from cuffed animals administered tracer immediately prior to death (Fig. 14) was uniform and not greater than the intensity of autofluorescence shown above.

## Discussion

Uptake of plasma macromolecules by the carotid artery wall of wild-type mice was elevated in regions close to the upstream and downstream ends of the tapered perivascular cuff, where lesions develop in apoE -/- mice fed a Western diet. Furthermore, uptake was more elevated upstream of the cuff (where lipid-rich lesions resembling the TCFA occur in hypercholesterolaemic mice) than downstream of it (where more fibrous lesions are seen). The data are consistent with the longstanding view that elevated uptake leads to the development of lesions and additionally support the novel hypothesis that the TCFA is triggered by an exceptionally high uptake, which is expected to lead to greater lipid accumulation.

The cuff was designed to restrict flow and hence produce low wall shear stress upstream of its wide end, and to induce recirculation and hence low and oscillatory wall shear stress downstream of its narrow end [8]. Consistent with this, our Doppler ultrasound data showed that the stenosis produced by the cuff was narrow enough to be flow limiting; lower blood velocities were observed upstream of the cuff than in the control vessel and a jet emerged from its throat. On this basis, elevated uptake appeared to occur in regions of low shear, with higher uptake in the low-steady region than in the low-oscillatory region.

CFD simulations agreed with the prediction of recirculation downstream of the cuff, but only when adjustment was made for depression of cardiac output by anaesthesia and/or inactivity; simulations based on the inflow velocities measured by Doppler ultrasound in inactive, anaesthetised mice, where velocities were <12 cm.s^−1^ (Re<11), did not show recirculation. Even at the highest adjusted velocities, the recirculation zone covered only 1/4 of the wall circumference and was only 250 µm long, and it was substantially smaller at lesser velocities. Furthermore, although the simulations showed that wall shear stress was lowered upstream of the cuff, as expected, it was not uniformly low near the cuff mouth, where uptake was high and lesions develop: a small region of *elevated* shear was seen at the entrance to the cuff because the vessel lumen narrowed, putatively as a result of remodelling caused by mechanical interference. Hence it may be incorrect to assume that uptake was elevated by low-steady and low-oscillatory wall shear stress. Indeed, it cannot even be assumed that the permeability patterns were caused by flow since the simulations showed a substantial pressure drop across the throat of the cuff with no recovery in the post-stenotic region. Effects of altered strain cannot be ruled out.

This view is reinforced by the pattern of uptake seen when the orientation of the cuff was reversed. Although the pattern of uptake was not reversed, which would have indicated that it entirely depended on the mechanical constriction produced by the cuff, the observed pattern could not be explained entirely by effects of flow either: uptake was elevated at the wider, downstream end of the reversed cuff, a region of entirely unremarkable flow in the corresponding CFD simulations. Hence it seems most likely that elevated uptake results from a mix of altered haemodynamic wall shear stress and altered mechanical stresses within the wall.

Some causes of elevated uptake can be ruled out. Thus we assume that stresses altered uptake by influencing transport properties of the wall rather than by influencing the degree of concentration polarisation at the luminal surface. (Concentration polarisation, akin to the formation of a filter cake, occurs when the flow of water into a porous medium is faster than the flow of particles contained within the water). That assumption is justified by the use of labelled albumin as a tracer; albumin does not concentrate significantly at the endothelial surface because its diffusion coefficient is sufficiently high that it can rapidly disperse from regions of poor convective mixing [29]. Note also that uptake in the middle of the cuffed region was not elevated, ruling out simple explanations based on the cuff enhancing permeability by injuring the wall or causing inflammation along its length. (The same is true for lesion prevalence, which is also low within the cuff [7]).

In previous studies, inhibiting NO production increased mean albumin uptake by the aortic wall in the vicinity of branch points in immature rabbits but not mature rabbits [12], [13]. In the present study, administration of L-NAME to uncuffed carotid arteries *decreased* the mean level of uptake. The inconsistency with earlier studies in the rabbit aorta adds to the complexity of the relation between vascular permeability and NO production, which is already an area of controversy [30]–[35]. More importantly, L-NAME abolished the difference in tracer uptake between regions upstream and downstream of the cuff, although uptake remained elevated in both regions. (Similarly, inhibiting NO synthesis abolishes differences in *in vivo* albumin uptake around branch points in the mature rabbit aorta [13].) Thus the particularly elevated uptake seen in the region pre-disposed to TCFA depends in some way on NO synthesis. Further investigations are required to determine whether this reflects a direct effect on transport properties of the wall, mediated through influences of NO on endothelial cells and/or smooth muscle cells, or an influence on mechanical strain arising from a pressor effect of NOS inhibitors. Additionally, although the cuff gives rise to an elevated level and non-uniform pattern of eNOS expression [8], a role for other NOS isoforms cannot currently be ruled out.

Two potential limitations of our study need to be evaluated. One is that uptake was assessed using a tracer based on albumin rather than on the large lipoproteins which deliver cholesterol to the wall. That was necessary because the level of labelled lipoprotein required to make high-resolution uptake measurements would render the mouse hyperlipidaemic, and hence might modify endothelial properties. Transport routes for macromolecules are size dependent and, if inferences can be made from studies in the microcirculation [36], not all albumin transport will take place via the route used by large lipoproteins. Despite this, studies in the aorta show good agreement between uptake patterns for albumin [9], [10] and low density lipoprotein [37] (the latter being transported by pathways independent of the LDL receptor [38]); and, as here, patterns of albumin uptake show an excellent spatial correlation with patterns of early lesion development [13]–[15].

The second potential limitation is that uptake was measured in wild-type mice rather than in the apoE -/- mice used to study effects of the cuff on plaque type. That was necessary in order to examine cuff-induced variations in uptake in the absence of early lesions, which are seen in apoE -/- mice of this age even on a normal diet [39]. Wild-type and apo E -/- mice are indistinguishable in their blood pressure, heart rate, and descending aortic velocity waveform but the knockout mice have a 60–70% elevation of mean and peak aortic blood velocity [40]. If anything, this elevation would be expected to increase rather than diminish differences between the upstream and downstream ends of the cuff because it would enlarge the recirculation zone – and hence the region of oscillatory flow – downstream of the cuff. We cannot exclude the possibility that the hyperlipidaemia occurring in apo E -/- mice alters their wall transport properties in some way; however, we have shown that patterns of aortic wall transport return to normal after a transient alteration when rabbits are administered a cholesterol-enhanced diet [41].

Hitherto, studies of arterial wall mass transport have been motivated by the concept that excessive uptake of circulating lipid-carrying macromolecules triggers the initiation of the fatty streak lesion characteristic of early atherosclerosis [42]–[45]. Studies focused on the development of the TCFA have instead considered the role of inflammatory processes, for example by looking at spatial variation of cytokine production during the development of lesions in the mouse cuff model [46]. The present data, obtained before lesions develop, are consistent not only with the long-standing view that elevated uptake triggers the development of fatty streaks but also with the novel hypothesis that excessively elevated uptake leads to the development of plaques resembling the TCFA. The excessive elevation reflects effects of altered fluid and solid mechanical stresses, and depends on endogenous NO synthesis.

The relevance of mouse models of plaque rupture to human disease has been extensively discussed elsewhere [e.g. 47, 48]; a particular concern is that not all aspects of the human TCFA are replicated. The cuff employed in the present study does appear to induce many relevant features when placed around the carotid artery of apoE -/- mice. Lesions produced at its upstream end have a lipid-rich core, a thin cap deficient in collagen and smooth muscle, high expression of matrix metalloproteinases and inflammatory markers, intraplaque hemorrhages and deposition of iron. If our results are applicable to spontaneous human as well as cuff-induced mouse disease, then controlling net wall uptake of macromolecules could provide a therapeutic pathway for reducing plaque rupture, and hence clinical events. Although methods for reducing uptake have not been established, suitable strategies might be obtained from further elucidation of the pathways studied here or from investigation *in vivo* of possibilities recently demonstrated *in vitro*, such as the barrier-tightening effects of sphingosine-1-phosphate on arterial endothelium [49] or the permeability-reducing effects of blocking endothelial cell apoptosis [50].

## Supporting Information

S1 File **Supporting table and figure.** Table S1. Velocities and Reynolds numbers used in CFD simulations. Peak systolic upstream velocities (V, cm/s) and corresponding Reynolds numbers (Re) for the cuff in the forward or reversed direction, as measured (“Ultrasound”) and after multiplying by factors to compensate for anaesthesia (“Conscious” – two different factors were used) and immobility (“Exercise”). These values were used in the CFD simulations. The ultrasound values were taken to indicate the centreline velocity and, assuming a parabolic flow profile, were halved to give the cross-sectionally averaged velocites (V) shown in the table. Figure S1. Hemodynamic wall shear stress (WSS) in cuffed vessels. A. Colour maps of WSS, obtained by CFD for different inflow velocities (V, cm/s), in carotid arteries with the cuff in the conventional direction. The throat of the cuff occurs at the narrowest part of the reconstruction. B and C. Detail of the throat region from two different viewpoints. D. Plot equivalent to (A) but for the cuff in the reversed direction.
(DOC)

S2. File **Supporting appendix.** Appendix S1. Effect of a Tapered Perivascular Cuff on Blood Pressure.
(DOCX)
