# Supplementary material for: Elevated Uptake of Plasma Macromolecules by Regions of Arterial Wall Predisposed to Plaque Instability in a Mouse Model
Source: PLoS One. 2014 Dec 22;9(12):e115728. doi: 10.1371/journal.pone.0115728 (PMC4274101; doi:10.1371/journal.pone.0115728)
Supplement: S1 File — Supporting table and figure. Table S1. Velocities and Reynolds numbers used in CFD simulations. Peak systolic upstream velocities (V, cm/s) and corresponding Reynolds numbers (Re) for the cuff in the forward or reversed direction, as measured (“Ultrasound”) and after multiplying by factors to compensate for anaesthesia (“Conscious” – two different factors were used) and immobility (“Exercise”). These values were used in the CFD simulations. The ultrasound values were taken to indicate the centreline velocity and, assuming a parabolic flow profile, were halved to give the cross-sectionally averaged velocites (V) shown in the table. Figure S1. Hemodynamic wall shear stress (WSS) in cuffed vessels. A. Colour maps of WSS, obtained by CFD for different inflow velocities (V, cm/s), in carotid arteries with the cuff in the conventional direction. The throat of the cuff occurs at the narrowest part of the reconstruction. B and C. Detail of the throat region from two different viewpoints. D. Plot equivalent to (A) but for the cuff in the reversed direction. (DOC) [file pone.0115728.s001.doc]

**Supplementary Material**

**Table S1. Velocities and Reynolds numbers used in CFD simulations.**

|  | Ultrasound | Conscious | | Exercise | |
| --- | --- | --- | --- | --- | --- |
|  |  | x1.05 | x1.72 | x1.05 | x1.72 |
|  |  |  |  | x1.88 | x1.88 |
| Forward V | 6.8 | 7.1 | 11.7 | 13.4 | 22.0 |
| Forward Re | 6.5 | 6.9 | 11.2 | 12.9 | 21.2 |
| Reverse V | 8.9 | 9.3 | 15.3 | 17.6 | 28.8 |
| Reverse Re | 8.3 | 8.8 | 14.4 | 16.6 | 27.1 |

**Supplementary Figure S1. Hemodynamic wall shear stress (WSS) in cuffed vessels**

S1A

S1B

S1C

S1D
