# Supplementary material for: Elevated Uptake of Plasma Macromolecules by Regions of Arterial Wall Predisposed to Plaque Instability in a Mouse Model
Source: PLoS One. 2014 Dec 22;9(12):e115728. doi: 10.1371/journal.pone.0115728 (PMC4274101; doi:10.1371/journal.pone.0115728)
Supplement: S2. File — Supporting appendix. Appendix S1. Effect of a Tapered Perivascular Cuff on Blood Pressure. (DOCX) [file pone.0115728.s002.docx]

Appendix S1– Effect of a Tapered Perivascular Cuff on Blood Pressure

Ethan M. Rowland

Department of Bioengineering, Imperial College, London, UK

1. Pressure loss in uncuffed regions

The upstream pressure drop ($\Delta P$) in the uncuffed portion of the vessel can be explained by Poiseuille's equation for pipe flow,

|  | $\Delta P=\frac{8\mu LQ}{\pi r^{4}}$ | 1 |
| --- | --- | --- |

where $\mu$ is the fluid viscosity, $L$ is the pipe (vessel) length, $Q$ is the flow rate and $r$ is the pipe radius. The pressure loss arises from the shearing forces between lamina in the fluid.

This equation shows that pressure drop is directly proportional to flow rate but inversely proportional to $r^{4}$. Hence halving the radius of the pipe increases the resistance to fluid movement by a factor of 16, if $Q$ is kept constant. If flow velocity ($V$) is kept constant the increase is by a factor of 4.Therefore relatively small changes in vessel diameter lead to large changes in resistance and thus pressure drop.

Applying this to a length of the carotid artery just upstream of the cuff, assuming a non-tapering lumen of diameter *D*,

$\mu$ = 0.004kg/(m.s), $L$ = 0.0015m, $D$ = 0.355x10^-3^m, $V$ = 0.2199m/s

then $\Delta P$ = 335.02Pa = 2.55mmHg. This compares well with the 4mmHg observed in the simulation, given that our geometry linearly tapers to around 0.3mm over this distance.

2. Pressure loss at the cuff throat

A pressure drop is expected at the cuff throat due to the increase in kinetic energy. It can be estimated using the equation,

|  | $\Delta P=0.5\rho V_{D}^{2}\left( \frac{D^{4}}{d^{4}}-1 \right)$ | 2 |
| --- | --- | --- |

derived from Bernouilli’s principle, where $d$ is the vessel diameter at the throat, $D$ is the upstream vessel diameter and $\rho$ is blood density. Given the well contoured geometry, flow separation is minimal even at the highest inlet velocity considered and thus the pressure should recover to close to upstream levels, distal to the throat. However Bernouilli's equation takes no account of non-conservative energy changes, in particular the work done against viscous shear stresses. At the low Reynold's numbers ($Re$) in the mouse carotid, viscous forces dominate. Hence the pressure drop is primarily due to shear stresses within the fluid and will not recover downstream.

From Young *et al.* [A1], the pressure drop across an arterial stenosis during steady flow may be estimated from,

|  | $\frac{\Delta P}{\rho V^{2}}=\frac{K_{v}}{Re}+\frac{K_{t}}{2}\left( \frac{A}{a}-1 \right)^{2}$ | 3 |
| --- | --- | --- |

where $A$ is the upstream cross sectional area, $a$ is the stenosis cross sectional area, and $K_{v}$ and $K_{t}$ are experimentally determined coefficients. At low $Re$, when viscous forces dominate, the pressure drop must be linearly related to velocity, and at higher $Re$, when turbulence losses dominate, the drop depends on the square of the velocity. Given the limited flow separation in the cuffed mouse carotid, the influence of the second term in this equation is small. The dependence of our data on this first term is evident when plotting $\Delta P$ against inlet $V$ (Fig. 1).


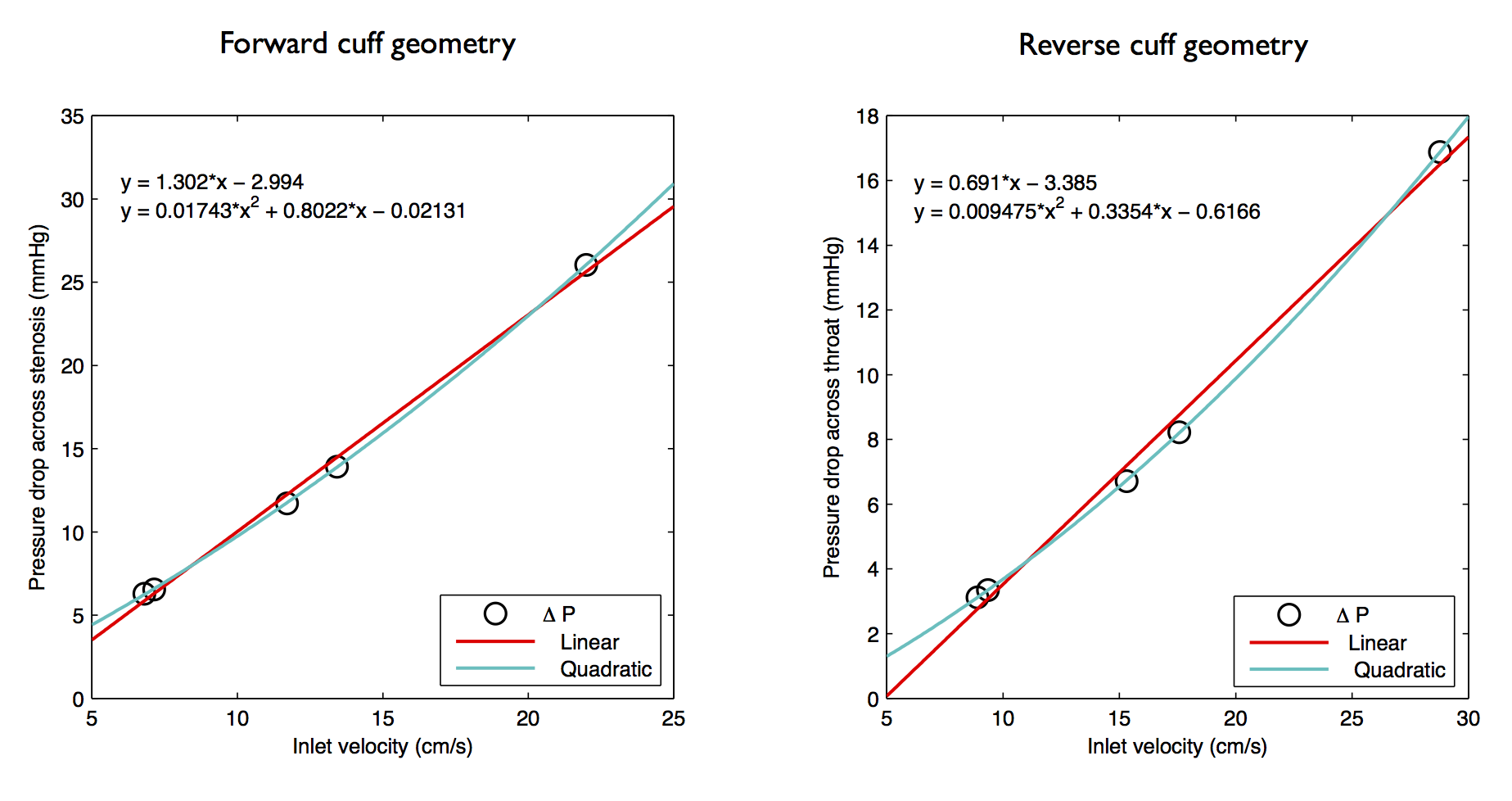


*Figure 1: Pressure drop across the stenosis against inlet velocity for the forward and the reverse cuff geometry. Simulated values are plotted and linear (red) and quadratic (black) fits applied to the data.*

$K_{v}$ and $K_{t}$ may be estimated from data fitting. Taking into account the units in Fig. 1, and using the quadratic fit and eq. 3, $K_{v}$ is 1000 for the forward cuff geometry and 410 for the reverse geometry. Young *et al.* [A1] showed that $K_{v}$ is strongly dependent on percentage stenosis rather than shape. The percentage area stenosis is ~ 50 for the forward geometry compared with 40 for the reverse (calculated using the area just upstream of the throat before the pressure drops). Seeley and Young [A2] showed that pressure drop is little affected by eccentricity of the stenosis; our values of $K_{v}$ for eccentric stenoses compare well with those of Young for symmetrical geometries.

Whilst a more severe stenosis should induce a larger pressure drop *in vivo* it is possible to have a smaller drop if the stenosis is flow limiting. Large pressure drops in vivo may be compensated for by decreases in peripheral resistance.

To summarise, the pressure drop is dependent on stenosis length (from eq. 1), strongly dependent on percentage area stenosis, largely independent of stenosis shape and eccentricity, and dependent on $Re$ only at low $Re$.

**References**

[A1] Young D.F., Cholvin N.R., Roth A.C., Pressure drop across artificially induced stenoses in the femoral arteries of dogs, Circ Res, 1975;36:735-743

[A2] Seeley B.D. and Young D.F., Effect of geometry on pressure losses across models of arterial stenoses, J Biomech, 1976;9:439-448
